# Wetting Behavior of a Three-Phase System in Contact with a Surface

**DOI:** 10.1021/acs.macromol.1c02559

**Published:** 2022-05-12

**Authors:** Biswaroop Mukherjee, Buddhapriya Chakrabarti

**Affiliations:** Department of Physics and Astronomy, University of Sheffield, Sheffield S3 7RH, U.K.

## Abstract

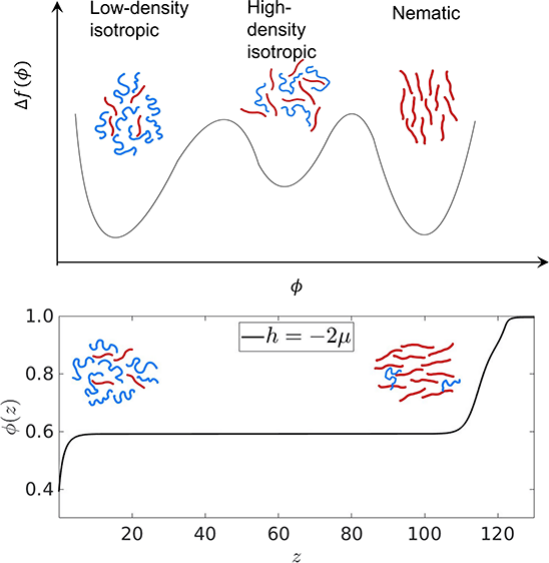

We extend the Cahn-Landau-de Gennes mean field theory of wetting in binary mixtures to understand the wetting thermodynamics of a three phase system (e.g., polymer dispersed liquid crystals or polymer-colloid mixtures) that is in contact with an external surface, which prefers one of the phases. Using a model free-energy, which has three minima in its landscape, we show that as the central minimum becomes more stable compared to the remaining ones, the bulk phase diagram encounters a triple point and then bifurcates and we observe a novel non-monotonic dependence of the surface tension as a function of the stability of the central minimum. We show that this non-monotonicity in surface tension is associated with a complete to partial wetting transition. We obtain the complete wetting phase behavior as a function of phase stability and the surface interaction parameters when the system is close to the bulk triple point. The model free-energy that we use is qualitatively similar to that of a renormalized free energy, which arises in the context of polymer-liquid crystal mixtures. Finally, we study the thermodynamics of wetting for an explicit polymer-liquid crystal mixture and show that its thermodynamics is similar to that of our model free-energy.

## Introduction

Wetting phenomena is ubiquitous in nature and arises in a variety of condensed matter systems ranging from classical fluids to superconductors and Bose–Einstein condensates.^1−4^ The most common example is a system having two bulk thermodynamic phases ϕ_α_^*b*^(*T*), and ϕ_β_^*b*^(*T*), in contact with a surface that prefers one of them. For such systems, the wetting behavior can be understood by two equivalent formulations: i.e. in terms of the (i) contact angle θ describing the geometric profile of a sessile drop of two coexisting bulk phases at a temperature *T* < *T_c_*, where *T_c_* corresponds to the bulk critical temperature, at the surface of a third phase,^1,3,5^ and (ii) profile ϕ(*z*), where ϕ corresponds to the concentration of the α/β phase as a function of the distance *z* from the surface of the third phase, which happens to be a spectator.^6^ In terms of the contact angle θ → 0, it signals a transition from a partial to complete wetting, while in Cahn’s approach, one has a macroscopic layer of one phase, ϕ_α_(*T*) in this case, residing at the surface, completely excluding the phase denoted by ϕ_β_^*b*^(*T*). A surface composition, ϕ_*s*_, an intermediate between the densities of the two coexisting bulk phases, ϕ_α_^*b*^(*T*) and ϕ_β_^*b*^(*T*), and decays smoothly to the bulk value of ϕ_β_^*b*^(*T*) is a characteristic of partial wetting. This transition from complete to partial wetting (also known as the interface unbinding transition) can be effected by lowering the temperature. Cahn showed that as one approaches the bulk critical point from below, the interfacial energy between the two phases goes to zero faster than the difference between their individual surface energies with the spectator phase. This thus necessitates a partial to complete wetting transition^6^ that has been well studied for small molecule mixtures.

The Cahn argument also applies to polymeric mixtures;^5,7−12^ however, there are two important differences. While for small molecule mixtures, the wetting transition occurs close to the bulk critical point, for polymer solutions, due to the low value of the interfacial tension between the immiscible phases, the transition occurs far from the bulk criticality.^8^ Second, unlike small molecule mixtures, one can study the wetting transition for polymers as a function of the molecular weights of the individual components. The complete to partial wetting transition is associated with lateral migration of material that results in interfaces being perpendicular to the confining wall.^13^ For a symmetric mixture of small molecules confined between asymmetric walls, (i.e., where one wall preferentially attracts a phase) Parry and Evans^14^ determined the concentration profile as a function of the confinement width and the temperature using the mean field theory. This formulation was extended to polymeric fluids under symmetric^15^ and asymmetric confinements.^16,17^

In all these situations, the bulk thermodynamics of the system is described by a mean field free energy with two minima corresponding to the stable phases of the system and a square gradient term, which accounts for the free energy cost associated with spatial variations.^12^ The surfaces prefer one of the phases and is modeled by a surface free energy that depends on the local density at the wall. The problem of minimization of the coupled bulk and the surface energies to obtain the concentration profiles can be mapped to a geometrical problem of Hamiltonian flow in phase space.^18^ Pandit and Wortis were the first to advocate the use of such phase portraits as a way of visualizing the solutions of the wetting profiles obeying appropriate boundary conditions.^18^

While the above discussion describes wetting in binary mixtures of simple or polymeric fluids, whose bulk thermodynamics is dictated by a free energy with two stable minimum at temperatures below a bulk critical temperature, there are many important physical situations where additional minima corresponding to locally stable phases may appear. Nematic ordering can induce additional local minima in the free-energy landscapes as the anisotropic interactions are known to play an important role in the problem of polymer crystallization.^19,20^ It is already known from theoretical^21^ and experimental investigations^22^ that consequences of interfacial phenomena is very subtle close to the bulk triple points even in one component systems and it leads to discontinuities in surface coverages owing to the first order nature of surface wetting. Additionally, residual elastic interactions in the matrix arising from the presence of cross-links are known to severely modify free-energy landscapes of bulk mixtures and thus affect surface migration and wetting behavior.^23^

In this paper, we present a consistent mean-field treatment of the thermodynamics of wetting for a two-component, three-phase system, which is in contact with an external surface, which acts as a spectator. The free energy of such a class of systems is modeled initially by two order parameters, (i) one distinguishing between the ordered and disordered phases and (ii) one that distinguishes between two disordered phases differing in density. We follow the Hamiltonian phase portrait method, in a semi-infinite geometry, to understand wetting for such a model, using a renormalized free energy, obtained by integrating out the order parameter that distinguishes between the high density disordered and ordered phases. The renormalized free energy is thus expressed in terms of a single order parameter corresponding to the relative density of the phases. We demonstrate that the stable solution for the surface fraction identified from the multiple solutions, which appear in the Cahn construction, corresponding to the one that minimizes the total surface free energy. We systematically vary the stability of the intermediate phase and the values of the surface interaction parameters and demonstrate the change in the nature of surface wetting transition as a result. Finally, we apply this scheme to study the wetting phase diagram of a model polymer dispersed liquid crystal^24,25^ described by a free energy, which accounts for both phase separation between low and high density polymer phases and the nematic ordering of the liquid crystalline component.

It is important to note that in the final section of this paper, we address the thermodynamics of wetting in a system where the mixture is a two component mixture (binary mixture) of polymers and orientable rods and this system is in contact with an external surface, which has a preferential affinity for one of the phases. The bulk binary mixture, in a certain parameter regime, exhibits three phases, and they correspond to a polymer rich isotropic phase, a nematogen rich isotropic phase, and a nematogen rich nematic phase, which shows broken orientational symmetry.^24,25^ The three minima in the case of polymer-dispersed liquid crystals arise due to the action of minimizing the nematic part (which initially has a second order parameter in the free-energy, see eqs 23 and 25) of the free-energy and plugging it back to obtain a “renormalized” free-energy, which is only dependent on one density and temperature. This renormalized free-energy, in a certain parameter regime, exhibits three minima, and the third would have been absent if the orientational degrees of freedom had not been accounted for in the free-energy. Additionally, if the anisotropic molecules also form a layered smectic phase at lower temperatures, then the renormalized free-energy would exhibit four minima in its landscape and when that system is coupled to an external surface, it would lead to an even richer wetting behavior.^26^ However, all these systems are binary mixtures of polymers and anisotropic, rigid molecules and thus a one order parameter description does suffice. Thus, our calculations would be more relevant for describing experimental systems like liquid crystalline surface coatings with switchable surface structures.^27−29^

The bulk thermodynamics of ternary or quaternary mixtures have been studied extensively; however, all these systems are mixtures of small molecules and they form rotationally isotropic phases.^30,31^ Other common examples of complex multi-phase systems are ternary amphiphiles,^32−35^ polymer–colloid mixtures,^36,37^ or metallic alloys.^38,39^ There has been a lot of recent interest in understanding the thermodynamics in ternary mixtures, where a description involving two order parameters is necessary for describing the bulk phase behavior.^40−43^ Depending on temperature and interaction parameters, several possibilities exist, e.g., one phase wets or spreads at the interface of the other two or the three phases may meet along a line of common contact with three non-zero contact angles. The transition between these two states is an equilibrium, three-phase wetting transition, and they appear in several varieties ranging from first to infinite order transitions.^41^

In the next section, we present the basic framework of the wetting calculations, which is followed by a section on the application of this method on the wetting transition in a simple binary polymer mixture. This is followed by a section on the wetting thermodynamics in the three-phase systems, and in the final section, we apply this formalism on a model polymer–nematic mixture.

## Wetting of a Binary Fluid in a Semi-Infinite Geometry

**Figure 1:**
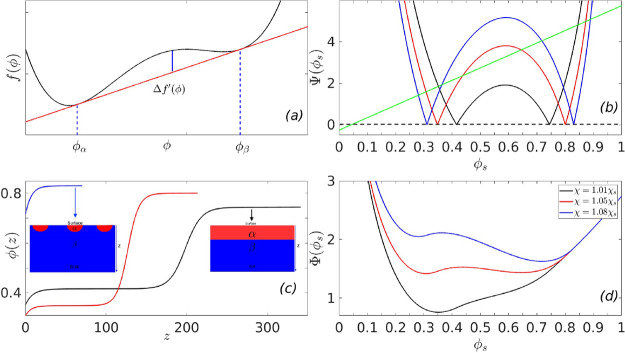
Schematic double minimum free energy with a common tangent in panel (a) (see eq 2). The Cahn construction associated with the wetting calculation for the binary polymer mixtures with surface energy parameters *h* = – 0.00026 and *g* = 0.006 is shown in panel (b). The concentration profiles for long polymers show a complete to partial wetting transition in panel (c). The effective surface free energy, obtained after minimizing the bulk thermodynamics of the system as a function of the surface fraction, is shown in panel (d).

The basic aim of the wetting calculation is to minimize the total surface free-energy functional,
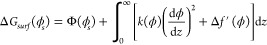
1where Δ*f*^′^(ϕ) is the bulk free energy contribution (after the common-tangent construction, see below),  accounts for the free energy cost arising from spatial gradients of the order parameter ϕ, with , and Φ(ϕ_*s*_)^12^ accounts for the surface free-energy of the external surface located at *z* = 0. The total free energy of the system incorporating bulk and surface contributions is denoted by Δ*G_surf_*(ϕ_*s*_). The bulk free-energy, Δ*f*^′^(ϕ), has a form that typically exhibits a single minimum at high temperatures, while it develops two distinct minimum at lower temperatures, corresponding to two bulk thermodynamic phases. The thermodynamic equilibrium corresponding to the same chemical potential and osmotic pressure among the two coexisting thermodynamic phases is ensured by a common-tangent construction,
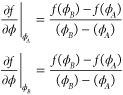
2where ϕ_*A*_ and ϕ_*B*_ are the two unknowns, which we identify as ϕ_α_(*T*) and ϕ_β_(*T*), with the convention, ϕ_α_(*T*) ≤ ϕ_β_(*T*). The free energy after the common tangent construction,
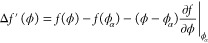
3enters the subsequent wetting calculations (see Figure 1a).

The minimization of the total free energy Δ*G_surf_*(ϕ_*s*_) (see eq 1) is done in two steps. First, the bulk contribution is minimized as a function of ϕ with the appropriate boundary conditions, i.e., the local density at the external surface should be ϕ_*s*_. The functional form that minimizes the bulk contribution expressed in terms of ϕ_*s*_ is then substituted back in eq 1. As a result, Δ*G_surf_*(ϕ_*s*_), the right hand side of eq 1, becomes a function of the yet undetermined surface fraction, ϕ_*s*_. This function is again minimized with respect to ϕ_*s*_ to obtain the surface fraction, which then allows one to obtain the wetting profile.

We use this framework to study wetting transition in a variety of systems. The equilibrium profiles, ϕ(*z*), which minimize the Lagrangian density, *L*(ϕ, ϕ̇), (the integrand of the above equation) obey the Euler–Lagrange equations
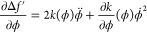
4where ϕ̇ =  and ϕ̈ = . The Hamiltonian can be obtained from the Lagrangian via a Legendre transformation given by
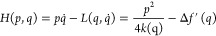
5where the coordinate *q* is ϕ and the conjugate momentum, *p*, is given by
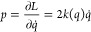
6

Since the Hamiltonian does not explicitly depend on *z*, it is a conserved quantity, which leads to the following equation
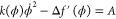
7where the constant of integration *A* = 0, since in the bulk, both Δ*f*^′^(ϕ) and ϕ̇ are zero. Thus, the minimal solution is given by
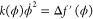
8which implies that the profile is given by,
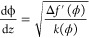
9

We take the positive sign of the root of eq 9 if ϕ < ϕ_∞_, as is the case for all calculations outlined in this paper. Substituting this solution into eq 1 allows us to change the integration variable from the spatial coordinate *z* to the density ϕ. As a result, we can rewrite eq 1 as
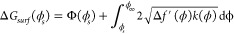
10

In this work, we discuss a situation where the low density phase is preferred by the surface, i.e., ϕ_*s*_ < ϕ_∞_ and we take the positive sign of the above square root. For ϕ_*s*_ > ϕ_∞_ only Φ(ϕ_*s*_) contributes to Δ*G_surf_*(ϕ_*s*_). In the final stage of the minimization scheme, we minimize Δ*G_surf_*(ϕ_*s*_), given by eq 10 with respect to ϕ_*s*_, to obtain the undetermined surface fraction. The surface free-energy used in this work is of the following form: , with *h* < 0 and *g* > 0. This choice makes the surface prefer a phase with ϕ_*s*_ = – *h*/*g*.

There are two ways to perform the final minimization, either by numerically computing Δ*G_surf_*(ϕ_*s*_) for various values of ϕ_*s*_ and then finding its minima or employing a Cahn-construction^6^ by equating the first derivative of eq 10 with respect to ϕ_*s*_ to zero, yielding
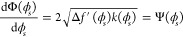
11

The surface fraction ϕ_*s*_ is then obtained from the intersection of the left and right hand side expressions of eq 11, numerically, which can result in multiple solutions, but the stable roots are found by comparison of areas. As discussed below, both these procedures yield the same value of surface fraction ϕ_*s*_.

The profile is obtained by integrating eq 9, which yields
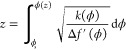
12

The boundary condition is obtained by substituting *z* = 0 in eqs 12 and 11 and taking their ratio, which finally yields
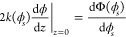
13

## Wetting Behavior of Binary Polymer Mixtures

As a simple example, we consider the complete to partial wetting transition, as the temperature *T* is deceased, in a binary mixture consisting of long (*N_A_* = 100) and short (*N_B_* = 50) polymers, in the presence of an external surface at *z* = 0, which prefers the short chain polymers (oligomers). The bulk thermodynamics is governed by a simple Flory–Huggins free energy^44^ of the form
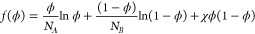
14where ϕ is the composition of polymers and (1 – ϕ) is the composition of the oligomers. The surface at *z* = 0 prefers the low ϕ component with the bare surface energy of the form
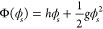
15where *h* < 0 and *g* > 0 are the surface parameters. We choose *h* = – 0.00026 and *g* = 0.006. This implies that (1 – ϕ), i.e., the oligomer composition, is supposed to be high near this surface. The bulk phase of the polymer mixture becomes unstable when χ is increased beyond the spinodal value χ_*s*_(ϕ_0_), where ϕ_0_ is the composition of the initially uniform mixture. The value of the Flory–Huggins χ parameter at the spinodal is given by,
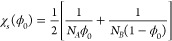
16

Figure 1 shows the transition from complete to partial wetting in a binary polymer mixture, in contact with an external surface, as the immiscibility parameter χ is systematically increased (or the temperature of the system is decreased, since χ ∝ 1/*T*). Panel (b) shows the Cahn construction for the Flory–Huggins free energy for χ = 1.01, (black), 1.05, (red) and 1.08χ_*s*_(ϕ_0_) (blue), where χ_*s*_(ϕ_0_) corresponds to the value of the immiscibility parameter at the spinodal (see eq 16). As shown, the Cahn construction yields multiple solutions, and the surface fraction, ϕ_*s*_, is chosen for which Δ*G_surf_*(ϕ_*s*_) is minimum (see panel (d)). This procedure is consistent with the area rule used for choosing the stable solution.^3^ The complete to partial wetting transition as the temperature is decreased is also evident from the change in the nature of the segregation profiles shown in panel (c) of Figure 1. At higher temperatures, i.e., for χ = 1.01 and 1.05χ_*s*_(ϕ_0_), the low ϕ bulk phase (i.e., oligomers) wets the external surface (ϕ_*s*_ < ϕ_α_) and completely expels the high density phase corresponding to polymers (see schematic in Figure 1c). When the temperature is decreased, i.e., for χ = 1.08χ_*s*_(ϕ_0_), a partially wetting profile, corresponding to ϕ_*alpha*_ < ϕ_*s*_ < ϕ_β_ is observed at the surface.

## Wetting in a Three-Phase System

While the bulk thermodynamics of binary polymeric mixtures always involves a free-energy with two local minima occurring at bulk densities, ϕ_α_(*T*) and ϕ_β_(*T*), complex mixtures with additional ordering fields, e.g., ternary amphiphiles,^33,35^ mixtures of nematics and polymers^24,25^ (we would be specifically discussing wetting in these systems later in this manuscript), can have free energies with additional metastable minima. The study of the influence of an ordering field on wetting transitions is very interesting with several technological applications in electro-optical devices^45,46^ and high modulus fibers.^47^

In this section, we extend the square-gradient mean field theory of wetting of a binary mixture to a three-phase system. In particular, we discuss the role of metastability on the wetting thermodynamics by studying a phenomenological form of free energy with three distinct local minima, whose location and relative heights can be varied. Since we do not have an explicit temperature-dependent free energy, we study the wetting transitions (i) as a function of the stability of the central minimum and (ii) by varying the surface parameters, *h* and *g*, which parametrizes Φ(ϕ_*s*_), the interactions of the external wall with the system. We focus on the Cahn-construction for a three-minima system and provide a criterion that dictates whether the wetting transitions are first order or continuous in nature. The three-phase free-energy that we consider has a piece-wise parabolic form
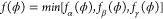
17where the *min* function chooses the minimum of three individual functions given by
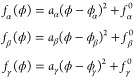
18with the following set of parameters: ϕ_α_ = 0.1, ϕ_β_ = 0.5, and ϕ_γ_ = 0.9, *a*_α_ = *a*_β_ = *a*_γ_ = 500 and the relative heights of the three minima are set by *f*_α_^0^ = 1, *f*_β_^0^ = 3.5, and *f*_γ_^0^ = 5, respectively.

**Figure 2:**
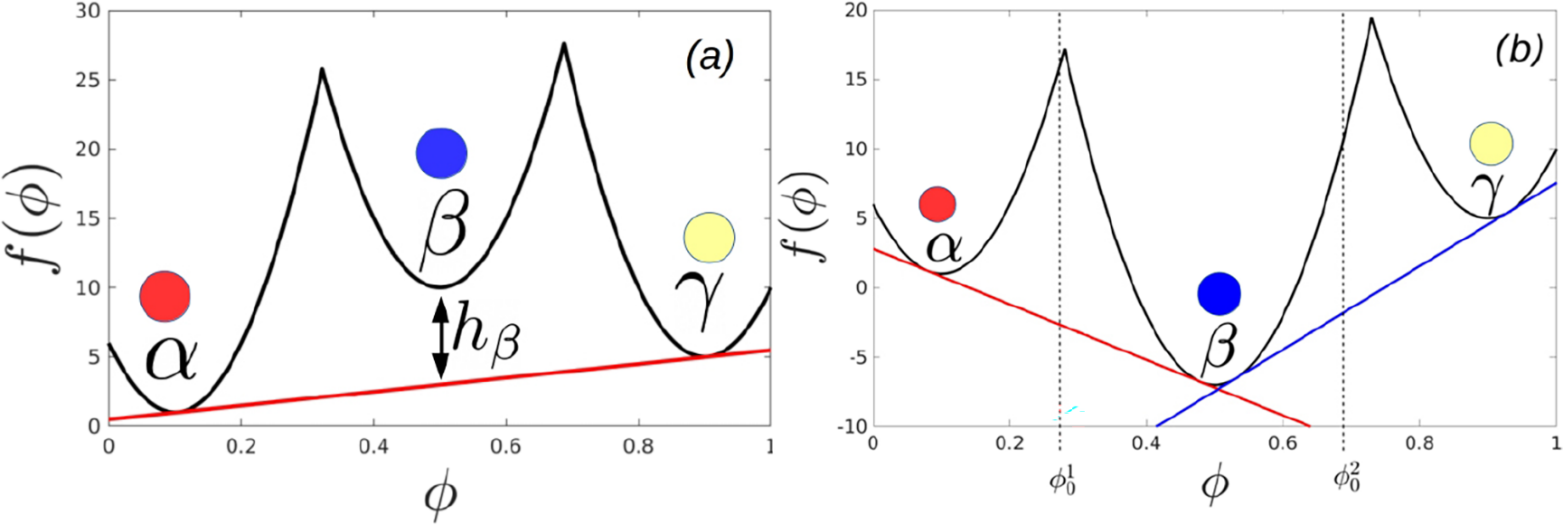
Triple-minimum free energy used for the calculation. The low (red), intermediate (blue), and high density (yellow) phases correspond to densities ϕ_α_ = 0.1, ϕ_β_ = 0.5 and ϕ_γ_ = 0.9 respectively. The variable *h*_β_ indicates the height of the barrier between the two thermodynamically stable phases between which the system splits.

We study the effects of the bulk thermodynamics on the wetting behavior by systematically varying the free energy parameters corresponding to intermediate values of ϕ i.e., *f*_β_^0^. As a result, the depth of the central minimum, *h*_β_, (see Figure 2) is varied systematically by changing *f*_β_^0^, such that −15 ≤ *f*_β_^0^ ≤ 10. The bare surface energy parameters are held fixed at *h* = – 0.3μ_*bulk*_ and *g* = – 12*h*, where μ_*bulk*_ corresponds to the slope of the red line in Figure 2a. Next, we study the wetting transition as a function of the surface parameters, i.e., *h* and *g*, close to the triple point (see red curve in Figure 4a).

**Figure 3:**
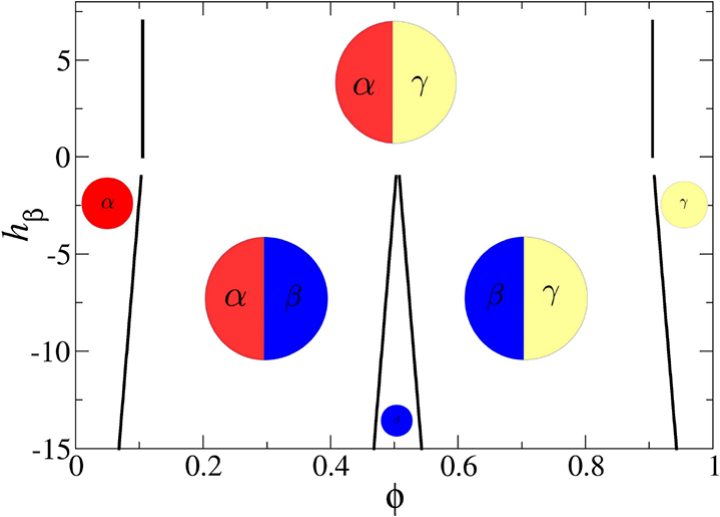
Phase diagram for the three minimum free energy as a function of the stability of phase β.

**Figure 4:**
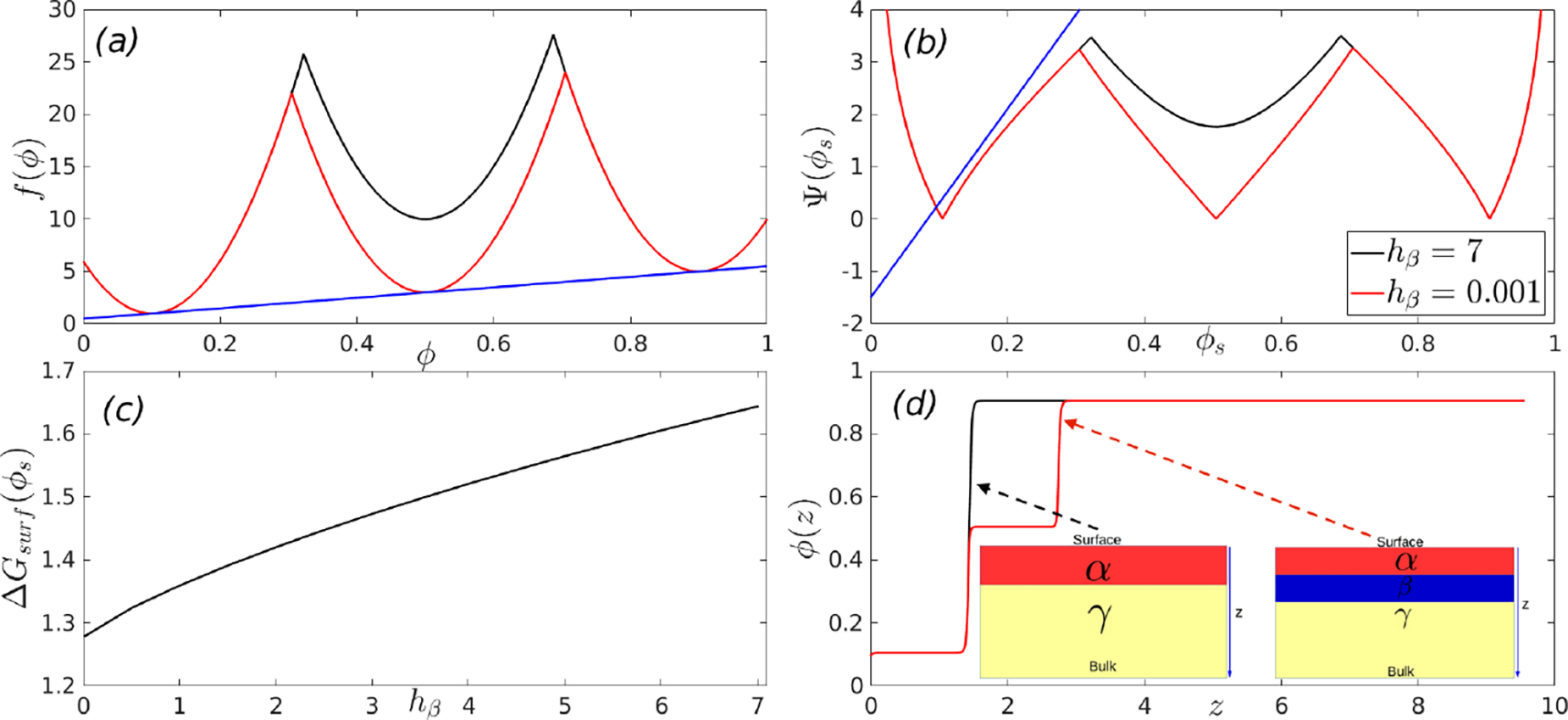
Wetting thermodynamics as function of *h*_β_, when it is positive and at the triple point. Panel (a) shows the free energies, panel (b) shows the Cahn constructions, panel (c) shows the dependence of the minimized surface free energy on *h*_β_, and panel (d) shows the order parameter profiles.

The bulk phase diagram of the three-phase free energy as a function *h*_β_ is shown in Figure 3, where each region is designated by a color of the phase/s that are stable in that region. For *h*_β_ > 0, the bulk free energy of a system, initially prepared with a uniform order parameter ϕ_0_, between ϕ_α_ and ϕ_γ_, is minimized by splitting between these two minima in a manner that preserves the initial order parameter value of ϕ_0_. Thus, the common tangent for the subsequent wetting calculation is drawn between the minimum at ϕ_α_ and ϕ_γ_ and the Δ*f*^′^(ϕ) for the subsequent wetting calculation should be constructed by subtracting off this common tangent from *f*(ϕ). Upon systematically decreasing *h*_β_ a situation arises when the minima of all three parabolic free energies lie on a common tangent (Figure 4a). This is the triple point when the three phases coexist simultaneously.

For *h*_β_ < 0, i.e., the β minimum corresponds to the most stable phase. If the initial composition is such that ϕ_0_ < ϕ_α_, a single phase with composition ϕ_α_ is chosen. When ϕ_0_ lies between the α and the β minima, the bulk free energy is minimized by the system splitting between these two phases with the corresponding fractions following the lever rule^44^ and the Δ*f*^′^(ϕ) for the wetting calculation has been constructed by subtracting off this common tangent from *f*(ϕ). In this regime, the γ component of the free energy does not enter the wetting calculations, as the α and the β minima have the lowest free energies according to our chosen parameters and hence the common tangent for the wetting calculation is drawn between these two states. The order parameter value, ϕ_∞_, deep in the bulk is a value close to ϕ_β_. For higher values of the initial composition, ϕ_0_, the β phase becomes the most stable phase. Upon increasing ϕ_0_ further, the bulk free-energy would be minimized when the system splits between the β and the γ minima and in this situation, the order parameter value deep inside the bulk, ϕ_∞_, would be close to ϕ_γ_.

Figure 4 shows the wetting thermodynamics as function of *h*_β_ > 0 and at the triple point, where the three phases coexist. Panel (a) shows the free energies corresponding to *h*_β_ = 7 (black) and h_β_ = 0.001 (red). We assume that the initial composition, ϕ_0_, lies between ϕ_α_ and ϕ_γ_. Thus, the bulk free-energy is minimized by the system splitting appropriately between ϕ_α_ and ϕ_γ_. We therefore draw a common tangent between these two minima, and the free energy, Δ*f*^′^(ϕ), which enters the wetting calculation is obtained by subtracting this common tangent from the free energy *f*(ϕ) (see eq 3). Panel (b) of Figure 4 shows the corresponding Cahn constructions *h*_β_ = 0.001,7. The derivative of the surface free energy, dΦ(ϕ_*s*_)/dϕ_*s*_, (blue line in panel (b)) intersects the curve  (RHS of eq 11), only at one point, which yields the surface fraction, ϕ_*s*_ < 0.1. The equilibrium value of the high-density phase corresponds to the material concentration deep in the bulk, ϕ_∞_ ≈ 0.9. Thus, these parameters set the lower and upper limits of integration for the expressions appearing in eqs 10 and 12.

Panel (c) shows the monotonically decreasing minimized surface free energy (the minimum of Δ*G_surf_*(ϕ_*s*_)), or the surface tension, as a function of *h*_β_ and panel (d) shows the order parameter profiles. From eq 10, it is clear that the surface tension has two contributions, one arising from the bare surface energy and the second from the area under the curve, . In this case, the surface fraction, ϕ_*s*_, is independent of thevariation in *h*_β_ and thus, while the bare surface energy remains unchanged the area under the curve, , monotonically decreases with *h*_β_. This leads to the monotonic decrease in surface tension with *h*_β_. A similar behavior has also been observed in calculations of surface tension in bulk systems with multiple minima in the free energy landscape.^48^ It is clear from panel (d) that away from the triple point, when *h*_β_ is positive and high, the order-parameter profile starts from ϕ_*s*_< 0.1 (α phase) and finally tends to its value of ϕ_∞_ ≈ 0.9 (γ phase) and the effect of the meta-stable β phase is negligible. Close to the triple point (see panel (d) of Figure 4) there is a split interface with the surface wet by the α phase thereby completely excluding the β and γ phases from the surface. The α phase is then wet by the β, which in turn is wet by the γ phase as one moves from the surface to the bulk. A similar behavior has already been observed for bulk ternary systems in the vicinity of the regime where the three phases formed by this system coexists.^49^ Schematic order parameter configurations for these two situations are shown in the insets in panel (d) of Figure 4.

**Figure 5:**
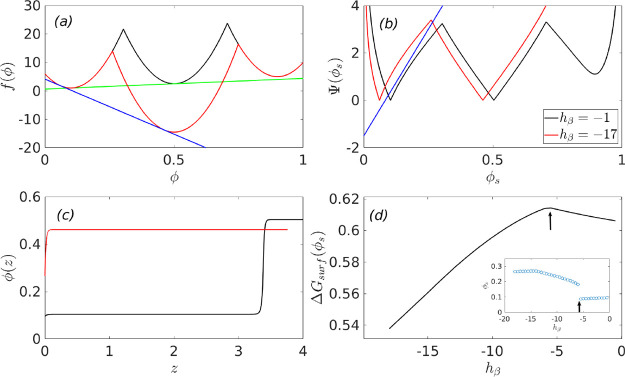
Wetting thermodynamics as function of *h*_β_, when it is negative and when the α and the β phases coexist. Panel (a) shows the free energies, panel (b) shows the Cahn constructions, panel (c) shows the segregation profiles, and panel (d) shows the dependence of the minimized surface free energy on *h*_β_. The inset to panel (d) shows the dependence of the surface fraction ϕ_*s*_ on *h*_β_, which signifies a transition from complete to partial wetting as one decreases *h*_β_.

Figure 5 summarizes the thermodynamics of wetting as a function of *h*_β_ when it is negative, and the initial composition, ϕ_0_, of the system is bracketed by ϕ_α_ and ϕ_β_ (see Figure 3 and the composition ϕ_0_^1^ marked in Figure 2b). Panel (a) of Figure 5 shows the free-energies at two representative values of h_β_ and the common tangents constructed between the free-energy minimum corresponding to ϕ_α_ and ϕ_β_. Thus, the relevant free energy Δ*f*^′^(ϕ), which enters the wetting calculation, is obtained by subtracting this common tangent from the free energy *f*(ϕ) shown in panel (a) of Figure 5. As a result, the value of the order parameter deep inside the bulk would be ∼ϕ_β_ = 0.5. As *h*_β_ becomes increasingly negative, the value of ϕ, in the vicinity of ϕ_α_, at which the common tangent between the α and the β minima intersects the free energy *f*(ϕ), decreases. This leads to an interesting behavior in the wetting phenomena. Panel (b) shows the Cahn construction for determing the surface fraction. The location where the line corresponding to  (blue line in panel (b)) becomes positive occurs at ϕ_*s*_ = −*h*/*g*. For small absolute values of *h*_β_, the value of ϕ_*s*_ at which  becomes zero (or Δ*f*^′^(ϕ_*s*_) becomes zero) is greater than ϕ_*s*_ = −*h*/*g*. This signifies a complete wetting of the surface by the α phase as shown in the order parameter profile, black line in panel (c). As *h*_β_ becomes increasingly negative, a situation arises when the value of ϕ_*s*_ at which Δ*f*^′^(ϕ_*s*_) becomes zero is less than ϕ_*s*_ = −h/g and this leads to a transition from complete to partial wetting and the red line in panel (c) yields a profile where the surface is partially wet by both the α and the β phases. This transition from complete to partial wetting results in a non-monotonic dependence of the surface tension or the minimized surface free energy, Δ*G_surf_*(ϕ_*s*_), shown in panel (d) of Figure 5. The value of *h*_β_ at which the non-monotonic behavior in Δ*G_surf_*(ϕ_*s*_) arises is that value where a transition from complete to partial wetting, of the surface by the α phase, occurs. This is shown in in the inset of panel (d), which shows the dependence of the surface fraction, ϕ_*s*_, on *h*_β_. This dependence of the surface tension is unlike what had been observed in the situation when *h*_β_ was positive.

**Figure 6:**
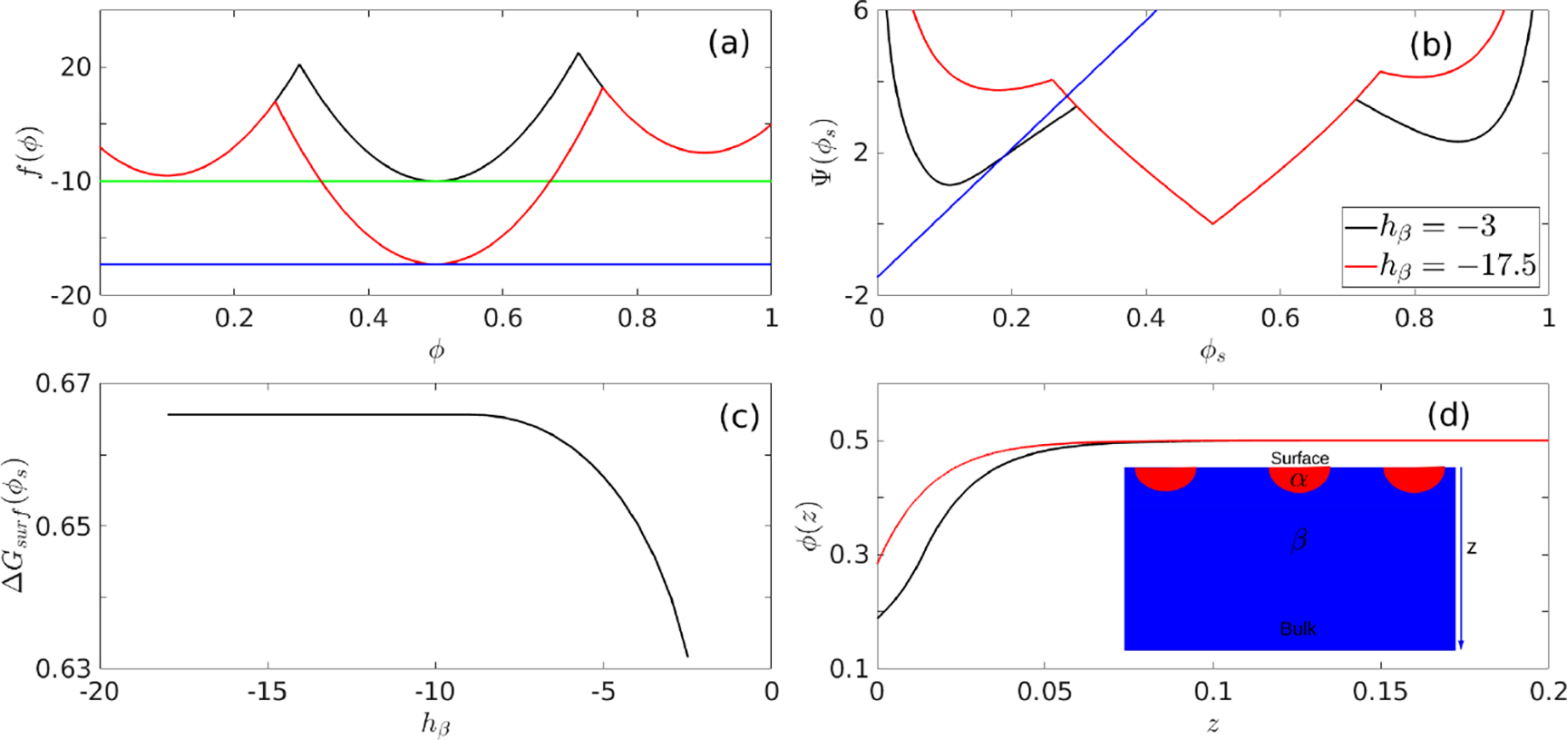
Wetting thermodynamics as function of *h*_β_, when it is negative, and the β phase is the most stable one. Panel (a) shows the free energies, panel (b) shows the Cahn constructions, panel (c) shows the dependence of the minimized surface free energy on *h*_β_, and panel (d) shows the segregation profiles.

For a negative h_β_, and ϕ_0_ ≈ ϕ_β_, the β minimum is the only stable state available, which minimizes the free energy of the system. In this situation, the reconstructed free-energy for the wetting calculation is obtained by drawing a horizontal tangent to the full free-energy at ϕ_β_ and subtracting this line from *f*(ϕ). The summary of the wetting calculation in this regime is presented in Figure 6, where panel (a) shows the free-energies and the horizontal tangent for two chosen values of *h*_β_. Panel (b) of Figure 6 shows the Cahn plots for obtaining the surface fraction, and in these situations, there is only one intersection between the red and black bulk contributions of  and the surface contribution arising from the  term and shown in blue. With decreasing *h*_β_, the value of surface fraction ϕ_*s*_ systematically increases (see the Cahn plots in panel (b) of Figure 6). Thus, in this situation, the two terms contributing to the surface tension in eq 10 has opposite dependence with decreasing *h*_β_. While the bare surface energy increases with ϕ_*s*_, the area under  decreases, with the bare surface energy contributing more, and this leads to the initial increase in the surface tension with decreasing *h*_β_ (see panel (c)). Once *h*_β_ falls below ∼−8, the surface line in panel (b) moves from the parabola corresponding to the α minimum to the one corresponding to the β minimum. After this point, the surface fraction remains invariant upon further decrease of *h*_β_ and as a result, the surface tension in panel (c) also shows a plateau. Panel (d) of Figure 6 shows the segregation profiles for two values of *h*_β_, and in both these situations, one observes partial wetting and the inset shows a two-dimensional, schematic representation of the order parameter profile.

If the initial composition, ϕ_0_, is bracketed by ϕ_β_ and ϕ_γ_, there are two possibilities for minimizing the bulk free energy, either (a) the ϕ_0_ is divided between the β and the γ minimum by order-parameter conservation and the minimum free energy is F_*A*_ for this situation or (b) the system tries to minimize its free-energy by splitting into the three minima and obviously conserving the order parameter and the minimum free energy is F_*B*_ for this situation. This second possibility arises as *f*(ϕ_α_) < *f*(ϕ_γ_). We prove below that F_*A*_ is always less than F_*B*_, which means that an initial uniform composition, ϕ_0_, which is between ϕ_β_ and ϕ_γ_, will always be split into order-parameter values obtained by drawing a common tangent between the β and the γ minima. The free-energy F_*A*_ is given by
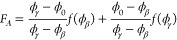
19

Is it possible to have a lower free energy with the order parameters partitioned between all the three free energy minima? To answer this, let us assume that we partition the initial order-parameter to all the three minima present in the free energy landscape, and then one can write the following equation owing to order parameter conservation constraint
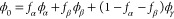
20

The above equation allows us to express the fractions *f*_α_ and *f*_γ_ in terms of the fraction *f*_β_
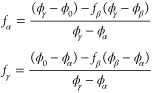
21

From the above fractions, one can write the free energy, where the initial order parameter has been partitioned into the three free energy minimum, in the following form
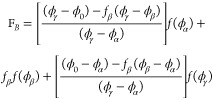
22

It is evident from the above expressions that *f*_γ_ > *f*_α_, owing to the choice of parameters for our model free-energy, and both of them linearly decrease as one increases *f*_β_, due to the constraint that their sum should be equal to unity. Thus, upon systematically increasing *f*_β_, *f*_α_ reaches zero first and this occurs when  and . At this point, the free energy of the system is *F_A_* and thus this proves that *F_B_* cannot be lower than *F_A_*, implying that when ϕ_β_ < ϕ_0_ < ϕ_α_, the lowest free energy would be obtained by splitting between β and the γ minimum. This thus implies that the relevant common tangent must be between the free-energy minimum at ϕ_β_ and ϕ_γ_ and the Δ*f*^′^(ϕ) should be constructed by subtracting off this common tangent from *f*(ϕ).

**Figure 7:**
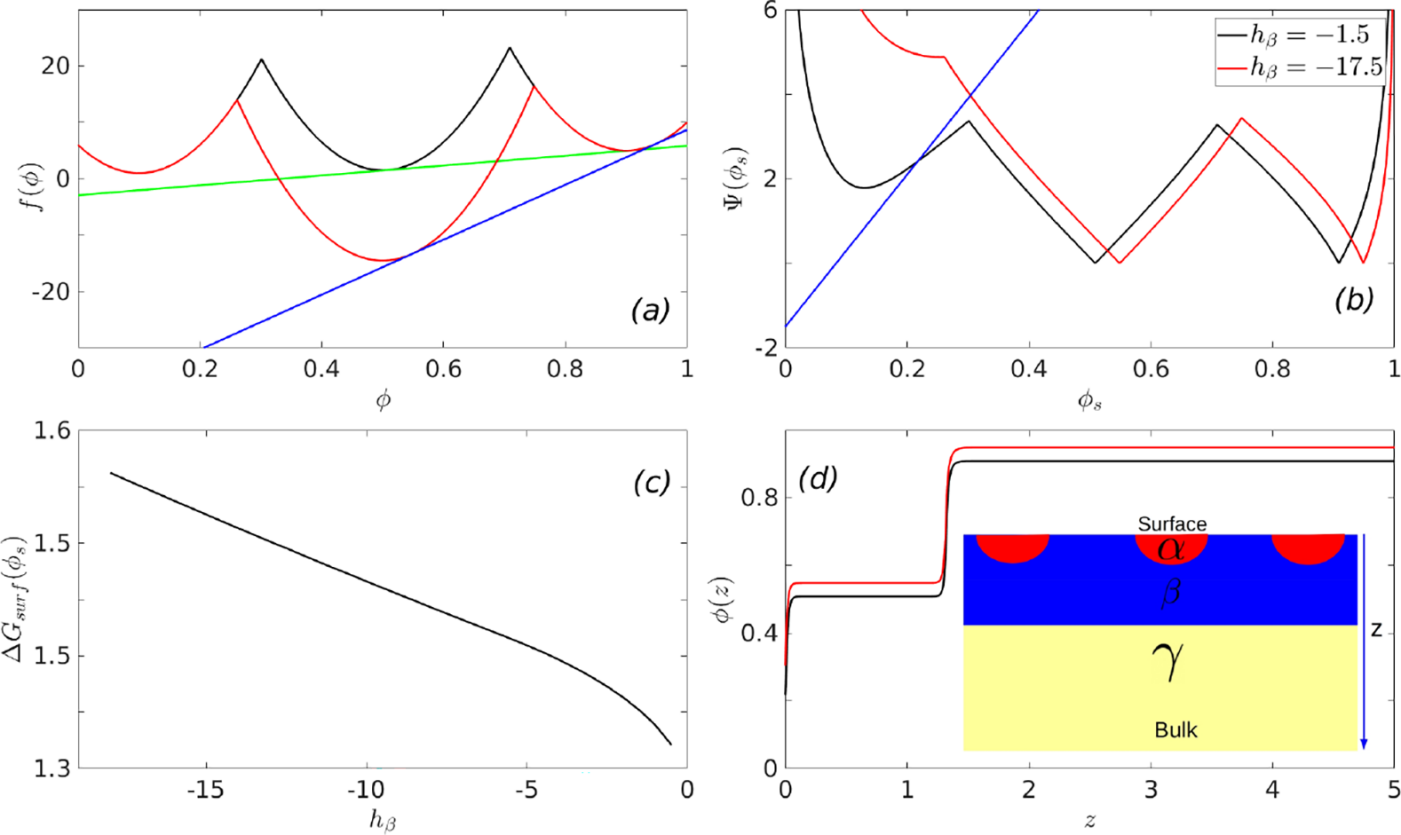
Wetting thermodynamics as a function of *h*_β_ when it is negative and when the β and the γ phases coexist. Panel (a) shows the free energies, panel (b) shows the Cahn constructions, panel (c) shows the dependence of the minimized surface free energy on *h*_β_, and panel (d) shows the segregation profiles.

Figure 7 summarizes the wetting thermodynamics for negative h_β_, when the initial composition ϕ_0_, is split between the ϕ_β_ and ϕ_γ_ minimum (the composition ϕ_0_^2^ in Figure 2b). Panel (a) of Figure 7 shows the free-energies and the common tangents, and panel (b) shows the Cahn plots yielding the surface fraction, ϕ_*s*_. Panel (c) shows the variation of the minimized surface free energy as a function of the decreasing *h*_β_, and panel (d) shows the segregation profiles for two values of *h*_β_. The inset to panel (d) shows a schematic, two-dimensional order parameter profile, which signifies that the surface is partially wetted by both α and β phases. In this situation, the minimized surface free energy, Δ*G_surf_*(ϕ_*s*_), increases with decreasing *h*_β_. This can be physically understood from the fact that the bare surface free energy is minimum for ϕ_*s*_≈ 0.083 and it increases for higher values of ϕ_*s*_. With decreasing *h*_β_, the value of ϕ_*s*_ increases, thus leading to a monotonic increase of the total surface free energy.

**Figure 8:**
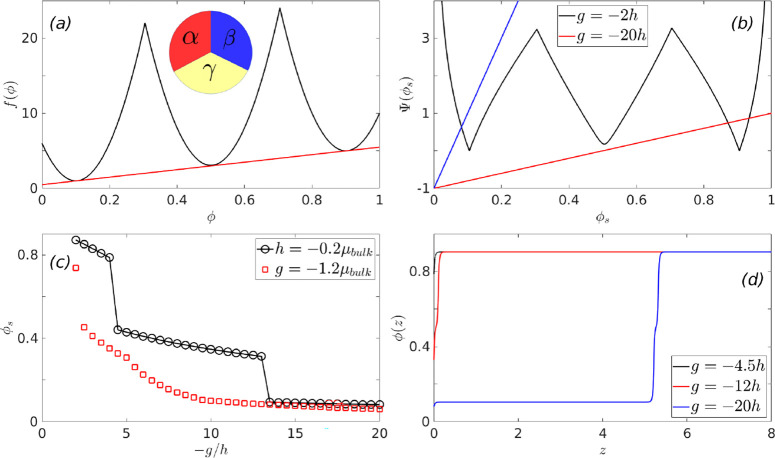
Wetting thermodynamics as function of the *h* and *g* parameters close to the triple point, where all the three phases coexist. Panel (a) shows the free energies (black line) and the common tangent in red, and panel (b) shows the Cahn construction, when *h* = −0.2 μ_*bulk*_ and corresponding to the smallest and the largest *g* values considered. Panel (c) shows the surface fractions as a function of parameter *g*, for *h* = −0.2μ_*bulk*_ (black line) and *h* = −1.2μ_*bulk*_ (red line). Panel (d) shows the order parameter profiles for three values of *g* corresponding to *h* = −0.2 μ_*bulk*_.

In the final set of calculations with the model three-minimum free energy, we compute the wetting phase diagram when the system is close to the triple point (where all three phases coexist) and vary the parameters, *h* and *g*, which parametrizes bare surface free-energy, Φ(ϕ_*s*_). Panel (a) of Figure 8 shows the triple-minimum free energy close to the triple point and a common tangent showing the coexistence of all the three phases. In these calculations, the value of the parameter *g* is varied systematically from *g_min_* = – 2*h* to *g_max_* = – 20*h*. The value of *h* is again varied between *h_min_* = – 0.2μ_*bulk*_ to *h_max_* = – 1.2μ_*bulk*_, where μ_*bulk*_ is the slope of the common tangent in panel (a). The corresponding Cahn plots for the lines , with the smallest and largest slopes are shown in panel (b), where *h* = – 0.2μ_*bulk*_. The surface lines correspond to , and thus *h* is the intercept of the surface line and *g* is its slope. In panel (c) of Figure 8, we observe that at a low absolute value of the parameter *h*, we observe two first order transitions (black line) for the surface fraction as a function of the parameter *g*, of which the first transition occurring at a value of (−*g*/*h*) ≈ 5 is between two partially wet states, whereas the transition occurring at (−*g*/*h*) ≈ 13 is a transition between partial to complete wetting states.

Upon increasing the absolute value of *h* (red line), the first order transition at occurring at higher value of *g* transforms to a continuous transition and also the jump in the surface fraction, ϕ_*s*_, occurring at low *g*/*h*, also decreases. The first order transitions occur when the line corresponding to the derivative of the surface free-energy, , cuts the curve  simultaneously at three values of ϕ_*s*_, and this only happens when the slope of the  line is small as in panel (b). When the magnitude of *h* increases, the  line never cuts the curve described by  simultaneously at three points and transitions tuned by varying parameter *g* become continuous in nature.^4^ Panel (d) of Figure 8 shows the order parameter profiles for the three values of *g*, when *h* is set to −0.2 μ_*bulk*_. At the highest absolute value *g* (blue line), we observe a complete wetting of the surface by the α phase. As the system is close to the triple point and as the common tangent simultaneously passes through all the three minima, the α phase at the surface is wet by the β phase and finally the γ phase emerges deep in the bulk. For lower values of the parameter *g* ≈ −12*h* (red line), one observes the β phase at the surface, which then leads to the γ phase in the bulk.

## Wetting of Polymer Dispersed Liquid Crystal Mixtures

As a real application of the results from the wetting calculation in a generic three-minimum free energy, we apply to the wetting thermodynamics of polymer dispersed liquid crystals. Here, we use as an example a model of PDLC previously studied by Matsuyama et al.^24,25^ for describing the bulk thermodynamics of a mixture of polymers and nematogens. A Flory–Huggins type free energy of the mixture, depending on two order parameters, is given by the free energy
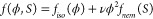
23

where ϕ is the composition of the nematic component, (1 – ϕ) is the composition of the polymer, and *f_iso_*(ϕ) is the Flory–Huggins-like isotropic part of the free-energy, given by
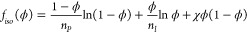
24where *n_P_* is the length of the polymer, *n_l_* is the length of the nematogens, and χ is the Flory–Huggins parameter controlling the thermodynamics of mixing. *f_nem_*(*S*) is the nematic part of the free-energy, with S as the nematic order parameter, which is given by,
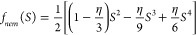
25where *η* is a factor dependent of the local nematic density ϕ, which couples the polymer and the nematic part of the free energy, appearing in the nematic free energy and is given by η = *n_l_*νϕ. Here, ν is a parameter controlling the isotropic to nematic transition and is given by
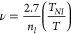
26

As a result of this, η is given by
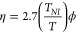
27

Similarly, χ, the parameter controlling the phase separation, is given by
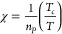
28

Thermodynamics dictates the minimization of the total free energy, toward which we proceed in two steps: first, we minimize the nematic part of the free energy and obtain a value of the nematic order parameter *S* (which is a function of η, which again is a function of ϕ). This *S* is then substituted back into the free energy, which now becomes a renormalized function of ϕ.

Upon minimizing *f_nem_*(*S*), we get the following equation for the non-zero roots,
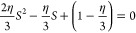
29

This equation has two roots, of which the positive (below *T_NI_* only the positive root contributes) is given by
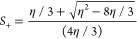
30

This root is now substituted back into the full free energy, which is now only a function of ϕ, and the thermodynamics of this model is derived form this modified free energy.

**Figure 9:**
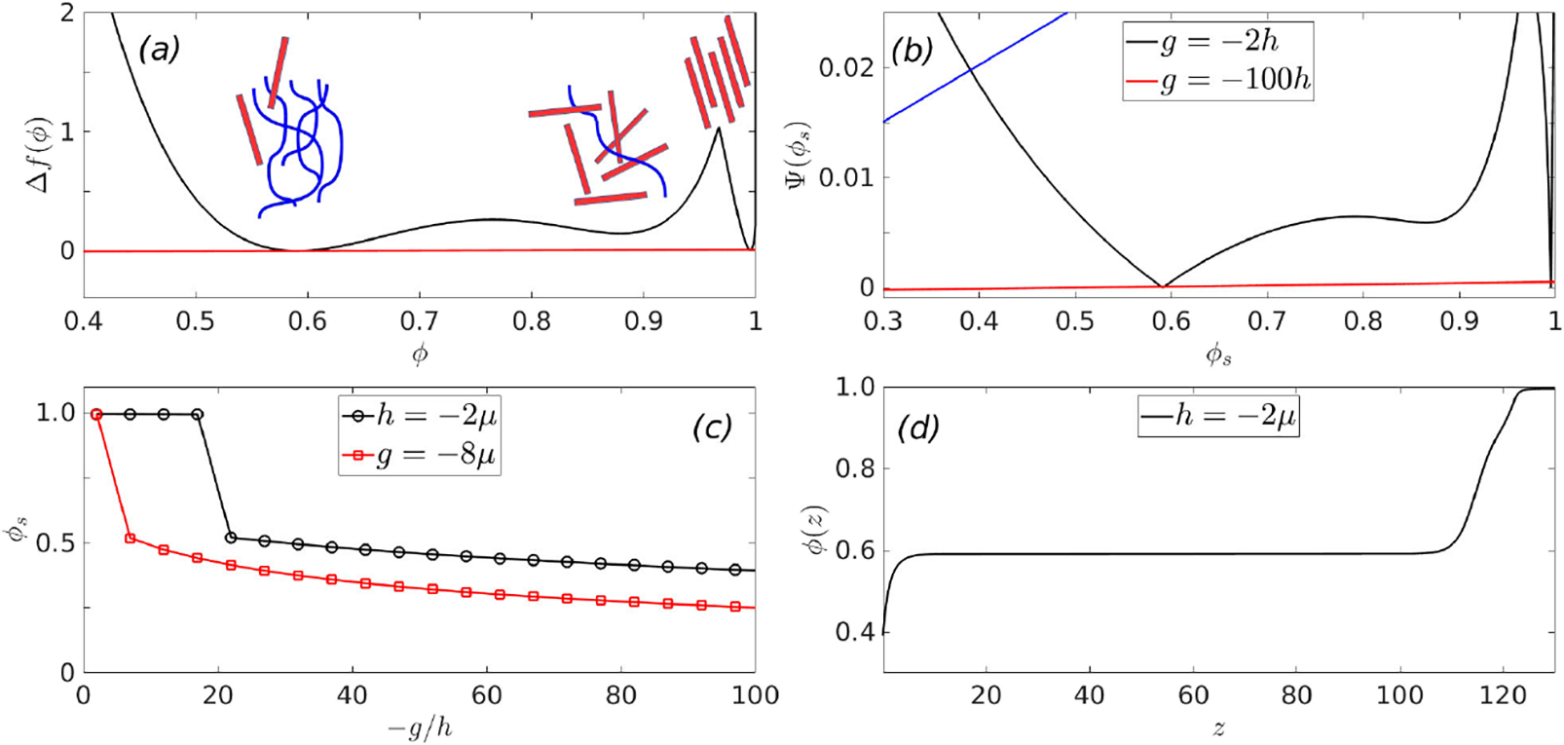
The renormalized free-energy is shown in panel (a) (after the minimization has been performed on the nematic part of the free-energy) as a function of the nematic volume fraction ϕ, showing the low density isotropic phases *I*_1_, the high density isotropic phase *I*_2_, and the nematic phase *N*. Panel (b) shows the Cahn construction, with the surface lines shown for the minimum and maximum *g* corresponding to *h* = −2μ. Panel (c) shows the variation of the surface fraction as a function of the parameter *g* (*h* = −2μ is shown in black, while *h* = −20μ is shown in red). Panel (d) shows the profile of the order parameter corresponding to the surface line shown in blue in panel (b).

We study a system for which *n_p_* = 20, *n_l_* = 2, and ν/χ = 3.1, and we are close to the triple point of the system at τ = 0.969, where the two isotropic phases, *I*_1_ and *I*_2_, and the nematic phase *N* are in coexistence. The bulk free energy or the free-energy difference of the system with respect to an initially homogeneous state, which enters the wetting calculation is given by,
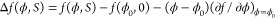
31where ϕ_0_ refers to the order-parameter of the initially homogeneous system, and its value is taken as 0.6 in the subsequent calculations. It is also assumed that the surface prefers the polymeric component characterized by the low value of the order parameter ϕ. This free energy is shown in panel (a) of Figure 9, which has three minima around ϕ ≈ 0.6 (isotropic), 0.88 (isotropic), and 0.99 (nematic). The parameters describing the surface interaction energy, , are the following: *g* is varied between −2*h* and −100*h*, where *h* is varied between −2μ and −8μ, where μ is the slope of the common tangent between the minima at ϕ = 0.6 and the one at ϕ = 0.88, in panel (a) of Figure 9. We observe qualitatively similar features in wetting behavior to our previously discussed model three-minimum free energy. Panel (b) shows the Cahn construction for the surface lines shown for the minimum and maximum *g* corresponding to *h* = −2μ. We observe in panel (c) that at a low absolute value of the parameter *h*, the surface fraction undergoes first order transitions (black line), as a function of the parameter *g*, while at higher absolute values of parameter *h*, one observes continuous transition in the surface fraction (red line). Panel (d) shows the profile of the order parameter corresponding to the surface line shown in blue in panel (b).

## Conclusions

We discuss a mean-field theory for the thermodynamics of wetting in complex mixtures, where there are three minima in the bulk free-energy landscape when exposed to a surface, which prefers one of the components. Such a free-energy landscape can arise in a variety of complex mixtures like polymer nematic mixtures, ternary amphiphiles, polymer-colloid mixtures, or metallic alloys. Interactions with the external surface are accounted via local potentials. We apply the Cahn-Landau-De Gennes mean field theory to understand the wetting thermodynamics of such a system as we systematically vary the height of the central minimum, and we find that the surface tension decreases monotonically with the height of this minimum, when it is unstable. As the central minimum becomes stable, the phase diagram bifurcates and we observe a non-monotonic dependence of the surface tension on the stability of the central minimum, in one of the branches, which is associated with a complete to partial wetting transition. In the other branch, we observe a monotonic increase in surface tension with an increasing stability of the central minimum. Close to the triple point, the wetting phase diagram computed by varying the bare surface energy parameters, *h* and *g*, yields two first order transitions in the surface fraction as a function *g* for low values of the parameter *h*. Upon increasing the absolute values of *h*, we observe that the first order transition in surface fractions gives way to continuous transitions. A geometric understanding of these phenomena is discussed. Finally, we present the wetting calculations for a polymer–nematic mixture, whose free energy actually has a three-minimum structure and show that the qualitative results obtained for our generic three-minimum free energy also holds for the polymer–nematic mixture.
